# Consultation Rate and Mode by Deprivation in English General Practice From 2018 to 2022: Population-Based Study

**DOI:** 10.2196/44944

**Published:** 2023-05-02

**Authors:** Emma Maria Vestesson, Kaat Lieve An De Corte, Elizabeth Crellin, Jean Ledger, Minal Bakhai, Geraldine M Clarke

**Affiliations:** 1 The Health Foundation London United Kingdom; 2 Great Ormond Street Institute of Child Health University College London London United Kingdom; 3 National Health Service England London United Kingdom

**Keywords:** primary care, deprivation, England, remote consultations, pandemic, COVID-19, CPRD Aurum, Clinical Practice Research Datalink, general practice, health inequalities, consultation, electronic health records, age, sex, care delivery, database, information management, population data, Index of Multiple Deprivation, IMD, cohort, longitudinal, data linkage, data link, general practice, random, consult, person-year

## Abstract

**Background:**

The COVID-19 pandemic has had a significant impact on primary care service delivery with an increased use of remote consultations. With general practice delivering record numbers of appointments and rising concerns around access, funding, and staffing in the UK National Health Service, we assessed contemporary trends in consultation rate and modes (ie, face-to-face versus remote).

**Objective:**

This paper describes trends in consultation rates in general practice in England for key demographics before and during the COVID-19 pandemic. We explore the use of remote and face-to-face consultations with regard to socioeconomic deprivation to understand the possible effect of changes in consultation modes on health inequalities.

**Methods:**

We did a retrospective analysis of 9,429,919 consultations by general practitioners, nurses, or other health care professionals between March 2018 and February 2022 for patients registered at 397 general practices in England. We used routine electronic health records from Clinical Practice Research Datalink Aurum with linkage to national data sets. Negative binomial models were used to predict consultation rates and modes (ie, remote versus face-to-face) by age, sex, and socioeconomic deprivation over time.

**Results:**

Overall consultation rates increased by 15% from 4.92 in 2018-2019 to 5.66 in 2021-2022 with some fluctuation during the start of the COVID-19 pandemic. The breakdown into face-to-face and remote consultations shows that the pandemic precipitated a rapid increase in remote consultations across all groups, but the extent varies by age. Consultation rates increased with increasing levels of deprivation. Socioeconomic differences in consultation rates, adjusted for sex and age, halved during the pandemic (from 0.36 to 0.18, indicating more consultations in the most deprived), effectively narrowing relative differences between deprivation quintiles. This trend remains when stratified by sex, but the difference across deprivation quintiles is smaller for men. The most deprived saw a relatively larger increase in remote and decrease in face-to-face consultation rates compared to the least deprived.

**Conclusions:**

The substantial increases in consultation rates observed in this study imply an increased pressure on general practice. The narrowing of consultation rates between deprivation quintiles is cause for concern, given ample evidence that health needs are greater in more deprived areas.

## Introduction

In the last few years, digital technology has enabled new ways of working in general practice. Before the COVID-19 pandemic, remote consultations (by telephone, video, text based, and web based) were steadily increasing on a background of supporting policy. The National Health Service Long Term Plan 2019 committed to every patient having the right to digital-first primary care by 2023-2024, and the 2019/20 general practitioner (GP) contract reform framework set out a requirement for all practices to offer web-based consultation systems by 2021 [1,2]. The COVID-19 pandemic served as a catalyst for the uptake of remote consultations, as guidance was issued to triage patient contacts, wherever possible, and encourage the use of remote consultations, if clinically appropriate, to avoid the risks of COVID-19 exposure to patients and staff [3]. Before the pandemic, it was reported that less than 30% of consultations were carried out remotely; within weeks, it increased to 77% [4].

Both remote and face-to-face consultation modes have advantages and disadvantages. Remote consultations can offer potential benefits through expanding access to services and appointment flexibility—in particular, for patients in rural areas, patients who find face-to-face consultations difficult, and those with substantial barriers for travelling to their general practice (eg, having mobility issues and employment or caring commitments). Faster access to care and a more cost-effective alternative to face-to-face appointments have also been highlighted as potential benefits to remote consultations [5]. In this way, remote consultations could decrease some of the current inequalities observed in the use of primary care.

On the other hand, there are concerns that remote consultations could exclude certain patient groups and exacerbate health inequalities [6]. Although the evidence around remote consultations and inequalities is limited, there is ample evidence that primary care is under more pressure in more deprived areas, so understanding the impact of a rapid increase in remote consultations is important [7]. Factors such as age, disability, digital exclusion, communication needs, data poverty, and lack of trust can impact people’s willingness or ability to access and benefit from remote consultations. A systematic review conducted at the start of the pandemic collated evidence on remote versus face-to-face consultations with a focus on inequalities and observed variations in the use of remote consultations, but the evidence was not conclusive [8]. A study of remote primary care for people experiencing homelessness during the pandemic found that the shift to remote consultations created barriers to accessing care due to factors such as the lack of funds to make calls or access to a telephone [9]. A cross-sectional study observed that there are differences in the proportion of consultations delivered remotely by category of the Index of Multiple Deprivation (IMD) [10]. However, a longitudinal study [5] did not observe a difference in the change of proportion of remote consultations over time by deprivation. Many of these studies are from before or the inception of the pandemic and the use of remote consultations has substantially changed. Studies from outside the United Kingdom do observe inequalities; but due to the difference in health systems, these results are unlikely to generalize to the English population [11,12].

In this study, we use person-level data from a large, nationally representative sample to describe contemporary patterns in consultation rates and modes in English general practice before and during the COVID-19 pandemic. We further investigate the inequalities in consultation rates and modes between individuals grouped by age, sex, and deprivation.

## Methods

### Study Design and Data

We performed a cohort study using person-level data from the Clinical Practice Research Datalink (CPRD) Aurum between January 2018 and February 2022. CPRD Aurum is a database with routinely collected data from general practices in England that use the EMIS Web information management system. CPRD contains data for over 40 million patients from 1332 practices in England as of May 2022 [13]. Patients are broadly representative of the English population based on age, sex, and deprivation [14]. CPRD provided linkage to the 2015 IMD at the patient level based on the patient’s Lower Layer Super Output Area and to the 2011 urban-rural classification based on the practice Lower Layer Super Output Area. The study protocol was approved by CPRD’s Research Data Governance (protocol number 21_000357).

### Ethics Approval

The study protocol was approved by CPRD’s Research Data Governance (protocol number 21_000357).

### Eligibility Criteria

We studied patients registered at general practices that participated in CPRD Aurum. Eligibility criteria were applied at both practice and patient level. A total of 400 practices located in England were sampled at random. Eligible patients were those with acceptable data quality (verified by CPRD); registered at one of the 400 practices at any point between January 2018 and May 2021; recorded as either male or female sex; and eligible for area-level linkage to the IMD 2015. From this cohort, 600,000 patients were randomly sampled. Three GP practices were identified by CPRD as having duplication issues, and therefore, were excluded. In addition, 2 patients were excluded as they no longer met the inclusion criteria after the final data set was extracted.

### Consultation Type and Consultation Mode

The primary information source on consultations was the CPRD Aurum “Consultation” table, which contains information on the type (eg, clinical or admin) and mode (eg, telephone or video) of the consultation. The consultation table can be linked to the “Staff” table to gain information about the staff member and the observations that occurred during the consultation [14]. We included consultations carried out by GPs, nurses, and other general practice care providers. Clinical consultations were identified. Consultations that were not attended were excluded. This builds on methods used on CPRD Gold and Aurum [15,16].

Consultations were further classified by mode of delivery as either remote (by telephone, video, SMS, and through the internet) or face-to-face (at the GP surgery or at home) consultations based on information in the consultation table for consultation mode or observations recorded during the consultation (Table S1 in Multimedia Appendix 1). Where a consultation’s mode was unclear, it was assumed to be face-to-face. A patient could have multiple consultations per day with a mix of modes. The final data set included consultations for patients between March 2018 and February 2022. For year-on-year comparisons, we grouped consultations in 12-month periods (March 2018-February 2019, March 2019-February 2020, March 2020-February 2021, and March 2021-February 2022), covering 2 years before the pandemic and 2 years during the pandemic.

### Statistical Analysis

We calculated person-years of observation over the study period based on the patient’s registration dates and when the practices submitted data to CPRD. Crude consultation rates and the proportion of remote consultations were calculated for each month and for 12-month periods. To compare consultation rates over time, the number of consultations, remote consultations and face-to-face consultations per patient were modelled using a negative binomial regression with person-years as an offset. The models included age, sex, deprivation, and pandemic year as well as interactions between these terms. A small number of patients without a deprivation quintile were excluded from the models.

From these models, we estimated adjusted consultation rates for the 12-month periods for patients grouped by deprivation quintile, age, and sex to allow absolute estimates of the differences between these groups. These rates were calculated for the “average” patient with respect to all the other variables in the model.

All analyses were carried out on a secure analysis server at the Health Foundation using R (version 4.0.3; R Core Team), with the ggeffects package for estimating predicted values [17,18].

## Results

### Consultation Rate

**Figure 1:**
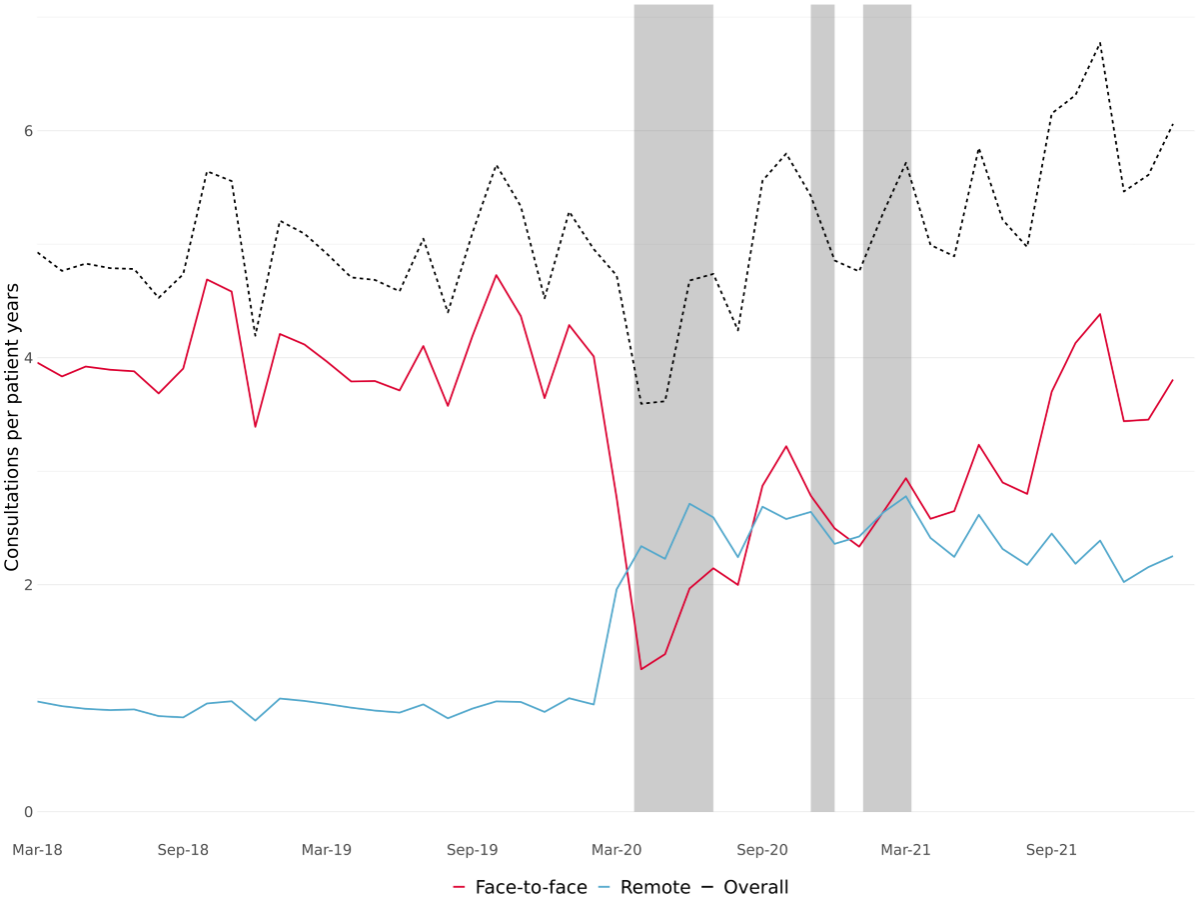
Overall consultation rates per patient-year and split by consultation delivery mode (ie, face-to-face and remote), calculated monthly between March 2018 and February 2022. The greyed-out areas correspond to periods of England-wide COVID-19 lockdowns.

The study included 9,429,919 consultations over 1,863,507 person-years of observation from 397 practices located across all regions in England between March 2018 and February 2022. Overall, face-to-face and remote consultation rates did not change materially between 2018-2019 and 2019-2020 (Table 1; Figure 1). Consultations averaged 4.92 and 4.94 per person-year in 2018-2019 and 2019-2020, respectively, with approximately 4 times as many face-to-face (4.00 and 4.02 per person-year in 2018-2019 and 2018-2020, respectively) as remote (0.91 and 0.92 per person-year in 2018-2019 and 2018-2020, respectively) consultations. During the pandemic, consultation rates initially dropped from 4.92 per person-year in 2018-2019 to 4.76 per person-year in 2020-2021; but by 2021-2022, it had increased to 5.66 per person-year, 15% higher than 2018-2019 (Table 1; Figure 1).

**Table 1:** Temporal trends in general practitioner (GP), nurse, and other health care professional (HCP) consultations by subtype of face-to-face and remote consultation modes. Crude rates are per person-year (N=1,863,507).

All GP, nurse, and other HCP consultations per person-year	2018-2019, crude rate (%)	2019-2020, crude rate (%)	2020-2021, crude rate (%)	2021-2022, crude rate (%)	Change between 2018-2019 and 2021-2022 (%)
All modes	4.92 (100)	4.94 (100)	4.76 (100)	5.66 (100)	15.0
Face-to-face	4.00 (81.3)	4.02 (81.4)	2.32 (48.7)	3.32 (58.7)	–17.1
Remote	0.91 (18.5)	0.92 (18.6)	2.45 (51.5)	2.34 (41.3)	155.3

### Consultation Mode

Over our study period, there were dramatic shifts in consultation modes. In 2020-2021, compared with 2018-2019, the rate of face-to-face consultations almost halved from 4.00 per person-year to 2.34 per person-year, while the rate of remote consultations more than doubled from 0.91 to 2.45 per person-year. During 2021-2022, rates of face-to-face consultations steadily recovered, whereas the rate of remote consultations remained stable; and by the end of our study period in February 2022, more consultations were delivered face-to-face (63%) than remotely (37%; Figure S1 in Multimedia Appendix 1). In the last year of our study, 2021-2022, rates of face-to-face consultations were 17.1% lower, and rates of remote consultations were 155.3% higher, compared with prepandemic 2018-2019.

### Variation by Age

Age-specific consultation rates had a consistently J-shaped distribution across all years with the highest rates in the youngest patients aged 0-4 years, decreasing to lowest levels in children aged 5-10 years, before steadily increasing in each age group to a peak in patients aged 75 years and older (Table 2).

**Table 2:** Consultations by mode and age per patient-years. All values for remote and face-to-face consultations are crude rates followed by percentages.

Age (years)	Consultations														
	2018-2019				2019-2020				2020-2021				2021-2022		
	Remote	Face-to-face	All modes^a^	Remote		Face-to-face	All modes^a^	Remote		Face-to-face	All modes^a^	Remote		Face-to-face	All modes^a^
0-4	0.95 (17.1)	4.61 (82.9)	5.56	0.95 (17)		4.64 (83)	5.60	1.96 (42.5)		2.64 (57.5)	4.60	2.3 (43.7)		2.96 (56.3)	5.26
5-10	0.4 (21)	1.49, (79)	1.89	0.39 (20.4)		1.51 (79.6)	1.89	0.87 (57.3)		0.65 (42.7)	1.51	0.98 (48.8)		1.03 (51.2)	2.00
11-17	0.38 (19.3)	1.58 (80.7)	1.96	0.37 (18.7)		1.62 (81.3)	2.00	0.99 (55.2)		0.8 (44.8)	1.79	1.08 (46.3)		1.25 (53.7)	2.33
18-24	0.63 (19.3)	2.63 (80.7)	3.26	0.63 (19.4)		2.63 (80.6)	3.26	1.83 (55)		1.5 (45)	3.33	1.76 (43.9)		2.25 (56.1)	4.01
25-34	0.73 (19.9)	2.94 (80.1)	3.67	0.72 (19.8)		2.93 (80.2)	3.66	2.03 (52.5)		1.83 (47.5)	3.86	1.91 (42)		2.63 (58)	4.53
35-44	0.74 (19.2)	3.12 (80.8)	3.86	0.74 (19.1)		3.13 (80.9)	3.87	2.07 (52.4)		1.88 (47.6)	3.94	1.98 (41)		2.86 (59)	4.84
45-54	0.84 (18.1)	3.81 (81.9)	4.65	0.86 (18.1)		3.92 (81.9)	4.78	2.44 (50.9)		2.35 (49.1)	4.79	2.35 (39.6)		3.59 (60.4)	5.95
55-64	0.97 (17)	4.72 (83)	5.69	0.98 (17)		4.8 (83)	5.78	2.81 (49.3)		2.89 (50.7)	5.70	2.64 (38.7)		4.18 (61.3)	6.82
65-74	1.29 (16)	6.76 (84)	8.05	1.32 (16.6)		6.65 (83.4)	7.97	3.55 (48.3)		3.8 (51.7)	7.35	3.27 (38.6)		5.21 (61.4)	8.48
≥75	2.5 (20.8)	9.52 (79.2)	12.02	2.51 (21.2)		9.34 (78.8)	11.84	5.92 (53.9)		5.06 (46.1)	10.99	5.06 (43.4)		6.61 (56.6)	11.67

^a^The percentage values are 100.

Between 2018 and 2020, consultation rates remained fairly steady within each age group with little variation in the proportion of consultations delivered remotely. In 2020-2021, a year-on-year decrease in the overall consultation rate was driven by those in the youngest (aged 0-17 years) and oldest (aged over 55 years) age groups. The decline was most pronounced in infants (aged 0-4 years), although this group sustained the highest proportion of face-to-face consultations (57.5%) during the first year of the pandemic.

In 2021-2022, consultation rates recovered to higher than prepandemic levels for all patients except for those in the youngest (aged 0-4 years) and oldest (aged ≥75 years) age groups. There was markedly less variation in consultation modes across the age groups in 2021-2022 compared with 2020-2021.

### Variation by Deprivation

**Figure 2:**
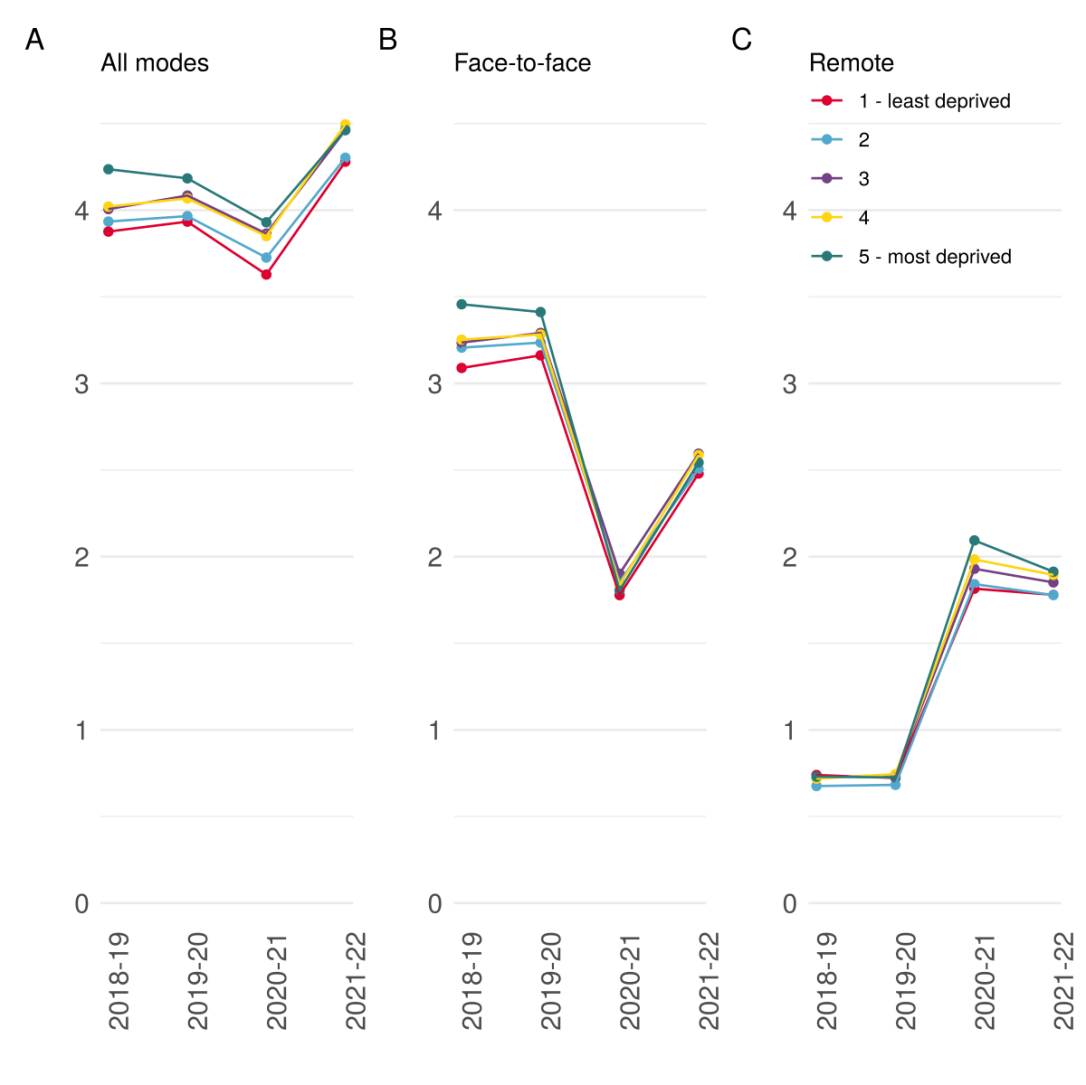
Yearly consultation rates stratified by deprivation quintiles. (A) Overall consultation rate per person per year. (B) Face-to-face consultation rate per person per year. (C) Remote consultation rate per person per year.

From the multivariable analysis, we present predicted age- and sex-adjusted consultation rates by deprivation quintiles over time (Figure 2 and Table S2 in Multimedia Appendix 1). Each quintile represents 20% of local areas in England.

Consultation rates increased with increasing deprivation in each study period and were at their highest in the second year of the pandemic (2021-2022) for all deprivation quintiles (Figure 2A). Over the study period, consultation rates increased by 10% in the least deprived quintile, compared with 5% in the most deprived quintile, effectively narrowing relative differences between deprivation quintiles.

Rates of face-to-face consultations also tracked deprivation quintiles, with patients living in higher deprivation quintiles consistently having a greater rate of face-to-face consultations than those living in lower deprivation quintiles (Figure 2B). Inequalities in rates of face-to-face consultations also narrowed during the pandemic with differences between the most and least deprived quintiles decreasing from 0.37 per person-year in 2018-2019 to 0.06 per person-year in 2021-2022.

There was little difference in the rate of remote consultations across deprivation quintiles before the pandemic (Figure 2C). During the pandemic, inequalities in rates of remote consultations widened, and patients in the higher deprivation quintiles had consistently higher rates than those in lower deprivation quintiles. In 2021-2022, remote consultation rates ranged from 1.91 per person-year in the most deprived quintile to 1.78 per person-year in the least deprived quintile.

**Figure 3:**
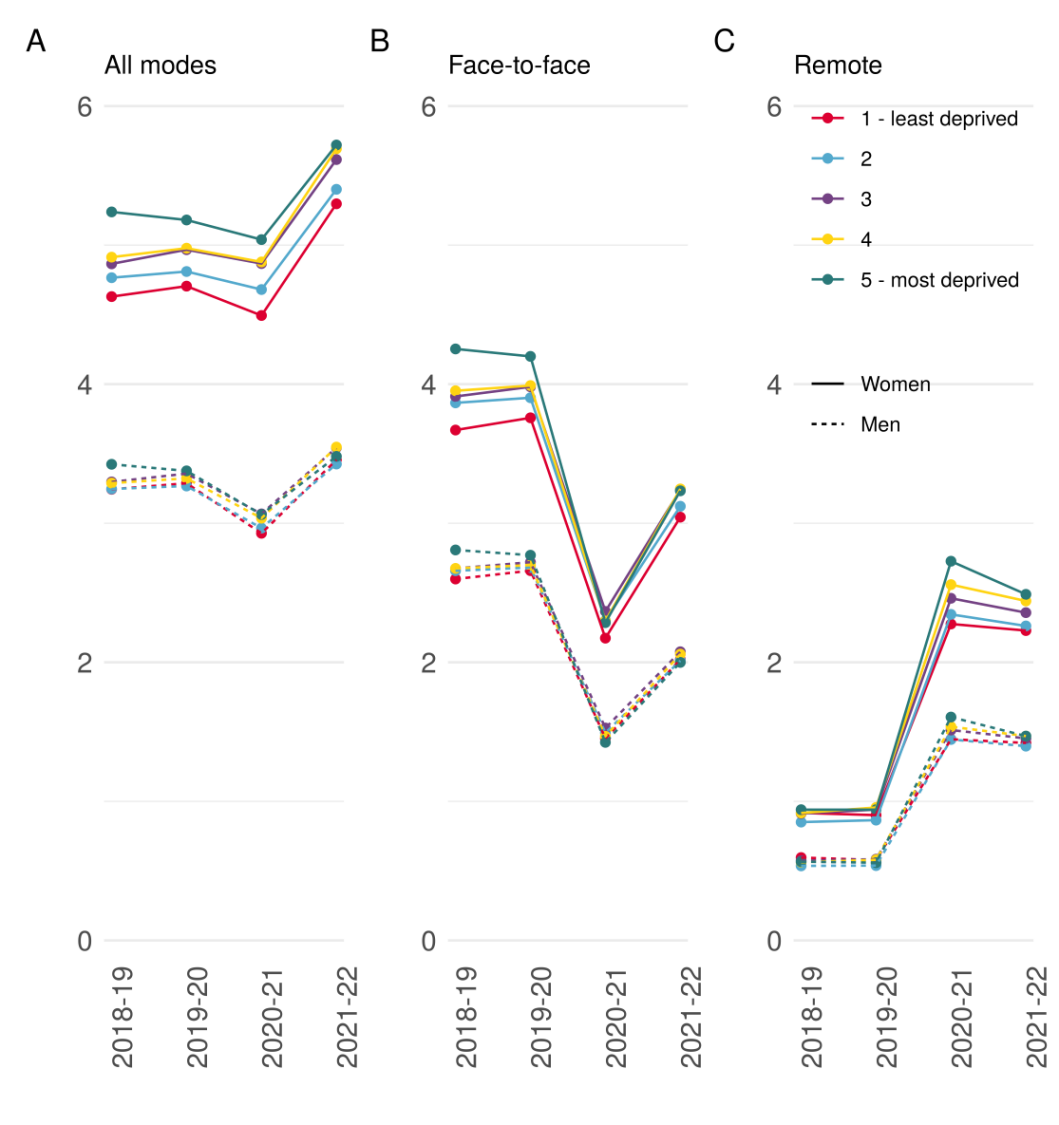
Predicted consultation rates over time by sex. (A) Overall consultations per year by deprivation. (B) Face-to-face consultations per year by deprivation. (C) Remote consultations per year by deprivation.

Figure 3 presents consultation rates by deprivation quintile and sex over time (Table S3 in Multimedia Appendix 1). Notably, men have consistently lower consultation rates than women. Trends in the rate of face-to-face and remote consultation rates across deprivation quintiles for women and men are similar to that observed for the overall population except that differences between the quintiles are narrower in men compared with women. For example, in 2018-2019, the difference in the consultation rate for men in the most deprived compared with the least deprived quintile was 0.18 per person-year, whereas it was 0.61 per person-year for women.

## Discussion

We provided contemporary, nationally representative data on the rate and mode of clinical consultations by GP, nurse, and other health care professional staff in general practice, analyzing trends across patient age, sex, and deprivation over 1 million patient-years. These are the first comprehensive data to break down consultations by mode. Consultation rates in primary cares were higher in 2021-2022 than before the pandemic; the pandemic has led to a dramatic reconfiguration of consultation modes in English general practices, with substantial heterogeneity across patient age, sex, and deprivation.

The consultation rates calculated in this analysis are lower than those observed by Hobbs et al [19] between 2007 and 2014. However, they are consistent with national data, and due to variations in methodology, direct comparisons can be difficult. Nevertheless, patterns across age and sex are consistent with those reported by Hobbs et al.

Before the pandemic, more than 75% of consultations were face-to-face. After a sharp drop at the start of the pandemic, the proportion of face-to-face consultations has slowly increased but remains below prepandemic levels at the end of our study. This reflects a significant shift in general practice processes with the adoption of a hybrid approach with a mix of appointment types. These trends in consultation modes have been observed elsewhere but not in a large nationally representative sample [5,8,9,20-22].

The dip in the proportion of face-to-face consultations at the start of the pandemic likely reflects operational guidance at the time. The pandemic created a drive for change [23]. Before the pandemic, the implementation of remote consultations was slow due to a combination of technological difficulties, confidence, and concerns about quality of care and low uptake from patients [24]. The model of general practice changed as a result, with more practices using doctor-led remote triage as the access point for services [4]. The subsequent increase in face-to-face consultations, despite repeated waves of high COVID-19 infection rates, likely reflects the embedding of processes to manage infection control risk, a better understanding of the risks of the virus, as well as changing government guidance on lockdowns and restrictions, although it is expected face-to-face consultation will remain below prepandemic levels to reflect new ways of working [25,26].

In the first year of the pandemic, the overall drop in the year-on-year consultation rates was driven by children (aged 0-17 years) and older patients (aged ≥65 years). COVID-19 restrictions, such as home schooling, social distancing, and shielding recommendations, reduced the spread of some diseases especially non–COVID-19 respiratory infections, which may be reflected in the lower consultation rates [27]. On the other hand, some age groups saw an increase in consultation rates in the first year of the pandemic, which could be COVID-19–related consultations. Despite the drop in overall consultations for these groups, they sustained higher proportions of face-to-face consultations. This reflects an active prioritization of groups by health need, as they are more likely to present with issues that are more complex or difficult to assess remotely.

We observed consistently higher consultation rates in patients living in more deprived compared with less deprived areas. This tallies with evidence that patients living in more deprived areas are more likely to have higher health needs [28]. The pandemic likely had a negative impact on everybody’s health; this is reflected in all quintiles of deprivation experiencing higher consultation rates in 2021-2022 compared to the prepandemic years. However, over the course of the pandemic, differences between the most and least deprived quintiles reduced by 5 percentage points, reflecting overall larger increases in consultation rates for patients living in the least deprived populations. During the pandemic, the health of patients in more deprived areas worsened faster compared with those patients in less deprived areas. Therefore, it seems unlikely that inequalities in health care needs have decreased over the pandemic.

Moreover, our findings may indicate that the demand-capacity gap has widened for patients living in more deprived compared to less deprived areas. Previous research highlighted that practices in more deprived areas manage 10% more needs adjusted for population size and receive 7% less funding adjusted for needs compared with practices in more affluent areas [29]. In addition, they faced greater workforce challenges with lower staff to patient ratios, lower recruitment rates, and more staff absences due to illness during the pandemic [7]. Overall, the evidence points to existing significant pressure limiting the capacity to stretch services further in response to pandemic- driven demand, exacerbating health inequalities. This indicates a need for greater support and investment in services in deprived areas.

At a patient level, reasons why patients in deprived areas had relatively fewer consultations during the pandemic may include a higher risk of being infected or getting severely ill with COVID-19 [30], a greater likelihood of lower levels of health and digital literacy [31], and an uneven economic impact [32]. These factors may have combined to result in both higher anxiety levels and greater concerns around contacting health care services due to risk of exposure to infection alongside greater difficulty in taking time off work to attend to health care needs. These findings correlate with reported patient overall experience of making an appointment where the fall in satisfaction rates was greater for practices in more deprived areas [33]. A greater reliance on technology could have also been a barrier to access for some patients in more deprived areas [34].

During the pandemic, the increase in remote consultations was reflected across all deprivation quintiles but was relatively larger for those living in the most deprived quintile—the difference in consultation rates between the most and least deprived populations increased by 13 percentage points for remote consultations in contrast to a decrease of 10 percentage points for face-to-face consultations. The reasons for the relatively greater use of remote consultations in more deprived populations during the pandemic are likely many and complex, relating to both practice- and patient-level effects. At the practice level, staff at practices in more deprived areas are more likely to be older or from ethnic minority backgrounds known to have been more impacted by COVID-19 [30,35-37]. Consequently, more staff might have been working from home in more deprived practices with more care being delivered remotely. At the patient level, socioeconomic variations in need could have played a role, as patients in more deprived quintiles are more likely to have long-term conditions and multimorbidity [38]. Patients living in deprived areas are both at higher risk of being infected and getting severely ill with COVID-19, which could lead to more remote consultations. Individual circumstances, such as ability to travel to the practice, could also impact the choice of modality [39].

Women having higher consultation rates than men has been widely reported [11]. Interestingly, sex differences in consulting rates varied by deprivation status, reflecting a greater socioeconomic gradient in consulting rates among women than men. This finding has been observed elsewhere [40] and could be a consequence of differences in the number of comorbidities between women and men, which also increases with increasing deprivation [41].

Digital technology will play an increasing role in general practice, and a deeper understanding of what mode of consultation suits particular patients’ needs and preferences is continuing to develop [42]. There is limited evidence on differences in clinical outcomes and patient satisfaction between remote and face-to-face consultations. However, a recent systematic review [43] suggests that overall system design, including the triage process and technology interface, is key to good outcomes especially staff workload, but more research is needed on a broad range of outcomes. Most remote consultations in England were telephone based and easier to access than other more technology-heavy remote consultation options, such as video calls. Despite the potential of digital tools to improve service delivery, it is vital to continuously improve their accessibility and usability and monitor the impact on health inequalities.

This study has several strengths. Using a rich clinical nationally representative patient-level data set that includes more than 1 million patient-years allows us to explore consultation modes by different patient groups. Comparisons between deprivation quintiles were adjusted for age and sex, making comparisons more accurate. We developed a novel method to derive consultation mode that combines information from the time of booking with what happened during the consultation to improve the accuracy of our results.

Data quality is one of the main limitations of this study, as the consultation mode is not consistently recorded in the data. The consultation mode was derived using a new method that has not been externally validated. The default assumption was that the consultation was face-to-face; we are therefore more likely to have underestimated the number of remote consultations. However, we found a strong increase in the use of remote consultations during the pandemic, with a peak around 75% of consultations being remote, which is consistent with other sources giving us confidence in our methods [14,16].

In summary, we found that general practice is delivering more consultations than ever, but that trends in consultation rates and modes across deprivation quintiles indicate exacerbation of existing socioeconomic inequalities. This general increase in the rate of consultations matches other evidence and reports from GPs, but further research is needed to ensure that consultation rates match health needs across deprivation groups.

## Appendix

Multimedia Appendix 1 Supplementary files.

## Abbreviations

CPRD: Clinical Practice Research Datalink
GP: general practitioner
IMD: Index of Multiple Deprivation
