# Supplementary material for: Consultation Rate and Mode by Deprivation in English General Practice From 2018 to 2022: Population-Based Study
Source: JMIR Public Health Surveill. 2023 May 2;9:e44944. doi: 10.2196/44944 (PMC10189615; doi:10.2196/44944)
Supplement: Multimedia Appendix 1 [file publichealth_v9i1e44944_app1.docx]

# Supplementary materials

Figure S 1 Percent of consultations that were remote


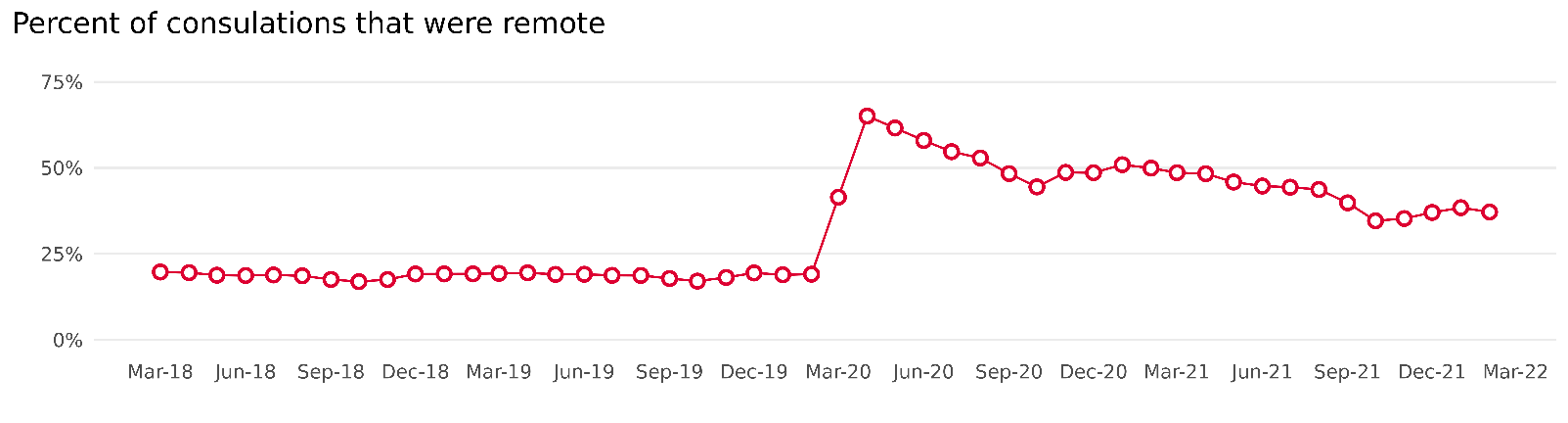


Figure S2 Percentage of remote and face-to-face consultations by age group


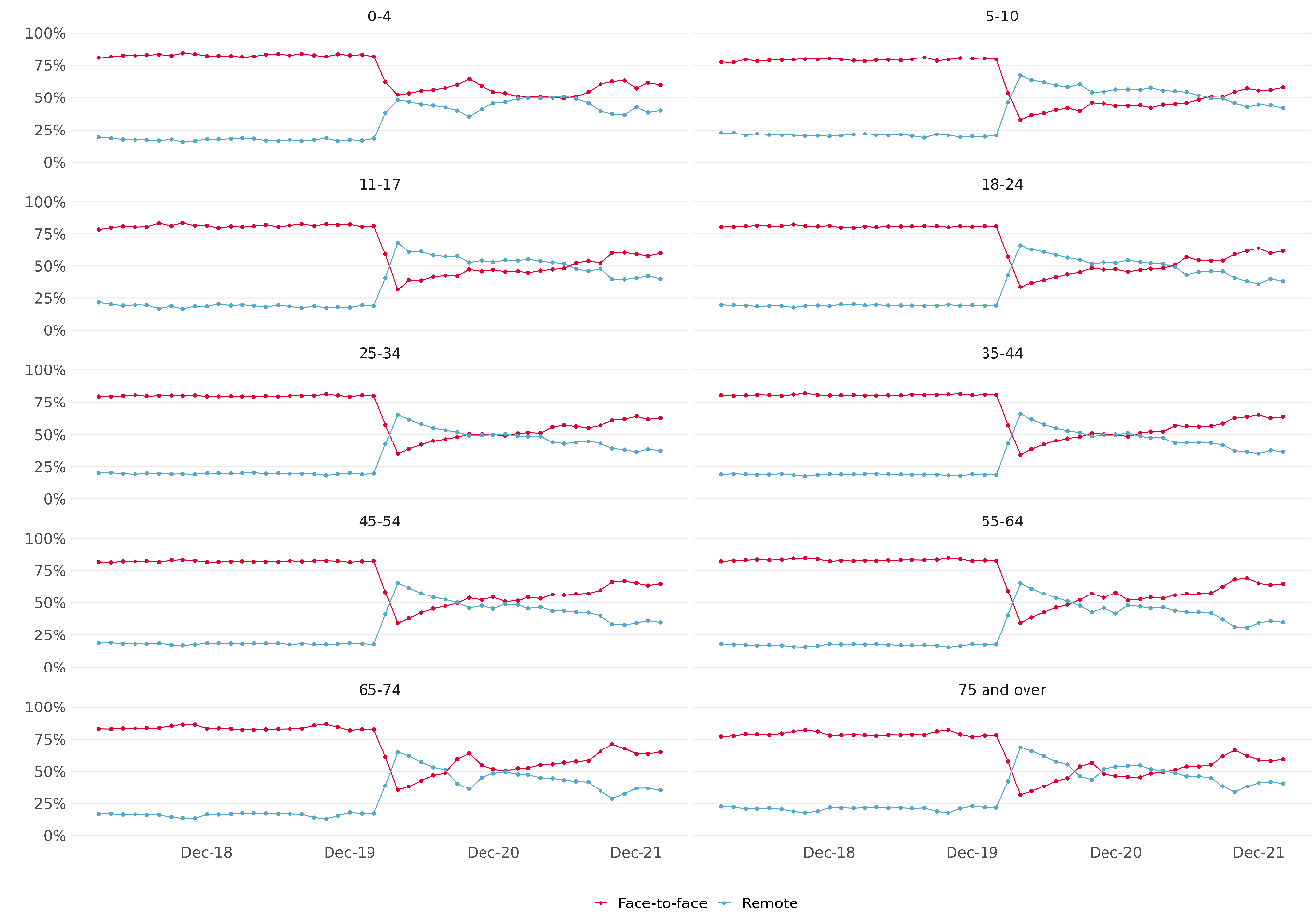


# Table S1 Codes to identify remote consultations from observations table

| obs_medcodeid | obs_medterm |
| --- | --- |
| 285327012 | telephone encounter |
| 1780456010 | telephone triage encounter |
| 62141000000119 | telephone call to a patient |
| 600831000000112 | consultation via multimedia |
| 62151000000116 | telephone consultation |
| 11990111000006100 | online triage encounter |
| 11990201000006100 | online triage outcome - video consultation |
| 12991091000006100 | advice given about 2019 novel coronavirus by telephone |
| 13012411000006100 | advice given about sars-cov-2 (severe acute respiratory syndrome coronavirus 2) by telephone |
| 7353351000006110 | telemedicine consultation with patient |
| 8012271000006110 | telephone consultation |
| 12991081000006100 | advice given about wuhan 2019-ncov (novel coronavirus) by telephone |
| 11639701000006100 | asthma nurse specialist telephone encounter |
| 11990141000006100 | online triage outcome - telephone consultation |
| 1778971000006110 | consultation via telemedicine web camera |
| 285332013 | encounter by computer link |
| 7786391000006110 | consultation via video conference |
| 8442071000006110 | econsultation via online application |
| 13012191000006100 | telephone consultation for suspected sars-cov-2 (severe acute respiratory syndrome coronavirus 2) |
| 600851000000117 | consultation via video conference |
| 1849991000006110 | first telephone consultation |
| 63381000000118 | telephone call from a patient |
| 8105181000006110 | consultation by telephone |
| 8344901000006110 | mental health prison inreach team telephone encounter |
| 12990411000006100 | telephone consultation for suspected 2019-ncov (novel coronavirus) |
| 12990421000006100 | telephone consultation for suspected wuhan 2019-ncov (novel coronavirus) |
| 1161431000000110 | telephone consultation for suspected swine flu |
| 8105301000006110 | assessment via video conference |
| 8361131000006110 | consultation via multimedia |
| 8099421000006110 | video-link encounter |
| 12990431000006100 | telephone consultation for suspected 2019 novel coronavirus |
| 12991071000006100 | advice given about 2019-ncov (novel coronavirus) by telephone |
| 11639691000006100 | clinical nurse specialist telephone encounter |
| 1958701000006110 | online communication |
| 1009181000006110 | telephone interpreter used |
| 480941000000110 | nurse telephone triage |
| 1480626017 | telephone follow-up |
| 11990121000006100 | online triage outcome |
| 1995221000006110 | email encounter |
| 1484972014 | patient given telephone advice during surgery hours |
| 1773301000006110 | e-mail consultation |
| 457040019 | patient given telephone advice out of hours |
| 11990211000006100 | online triage outcome - email sent to patient |
| 11990131000006100 | online triage outcome - prescription issued |
| 8105161000006110 | remote consultation |
| 1672891000006110 | telephone call to relative/carer |
| 661661000000111 | e-mail encounter to carer |
| 1672901000006110 | telephone call from relative/carer |
| 1778991000006110 | consultation via sms text message |
| 11990171000006100 | online triage outcome - patient to make appointment online |
| 285396014 | patient 'called' - prevention |
| 2015331000006110 | telehealth monitoring - telephone call to patient |
| 2015351000006110 | telehealth monitoring - telephone call to relative/carer |
| 1161531000000110 | advice given about swine flu by telephone |

Table S2

|  | 2018-19 | 2019-20 | 2020-21 | 2021-22 |
| --- | --- | --- | --- | --- |
| Overall | | | | |
| 1 - least deprived | 3.88 (3.84-3.91) | 3.93 (3.9-3.97) | 3.63 (3.6-3.66) | 4.28 (4.24-4.32) |
| 2 | 3.93 (3.9-3.97) | 3.97 (3.93-4) | 3.73 (3.69-3.76) | 4.3 (4.26-4.34) |
| 3 | 4.01 (3.97-4.04) | 4.08 (4.05-4.12) | 3.86 (3.83-3.9) | 4.46 (4.42-4.5) |
| 4 | 4.02 (3.99-4.05) | 4.07 (4.03-4.1) | 3.85 (3.82-3.88) | 4.5 (4.46-4.53) |
| 5 - most deprived | 4.24 (4.2-4.27) | 4.18 (4.15-4.22) | 3.93 (3.9-3.96) | 4.46 (4.43-4.5) |
| Face-to-face | | | | |
| 1 - least deprived | 3.09 (3.06-3.12) | 3.16 (3.14-3.19) | 1.78 (1.76-1.79) | 2.48 (2.46-2.5) |
| 2 | 3.21 (3.18-3.23) | 3.24 (3.21-3.26) | 1.85 (1.84-1.87) | 2.51 (2.48-2.53) |
| 3 | 3.24 (3.21-3.26) | 3.29 (3.26-3.32) | 1.9 (1.88-1.92) | 2.59 (2.57-2.62) |
| 4 | 3.25 (3.23-3.28) | 3.28 (3.26-3.31) | 1.84 (1.82-1.85) | 2.59 (2.56-2.61) |
| 5 - most deprived | 3.46 (3.43-3.48) | 3.41 (3.38-3.44) | 1.8 (1.79-1.82) | 2.54 (2.52-2.56) |
| Remote | | | | |
| 1 - least deprived | 0.74 (0.73-0.75) | 0.72 (0.71-0.73) | 1.81 (1.8-1.83) | 1.78 (1.76-1.8) |
| 2 | 0.68 (0.67-0.68) | 0.68 (0.67-0.69) | 1.84 (1.82-1.86) | 1.78 (1.76-1.8) |
| 3 | 0.72 (0.71-0.73) | 0.74 (0.73-0.75) | 1.93 (1.91-1.95) | 1.85 (1.83-1.87) |
| 4 | 0.72 (0.71-0.73) | 0.74 (0.74-0.75) | 1.98 (1.96-2) | 1.89 (1.87-1.92) |
| 5 - most deprived | 0.73 (0.72-0.74) | 0.73 (0.72-0.73) | 2.09 (2.07-2.11) | 1.91 (1.89-1.93) |

Table S3

|  | 2018-19 | | 2019-20 | | 2020-21 | | 2021-22 | |
| --- | --- | --- | --- | --- | --- | --- | --- | --- |
|  | Women | Men | Women | Men | Women | Men | Women | Men |
| Overall | | | | | | | | |
| 1 - least deprived | 4.63 (4.58-4.68) | 3.24 (3.21-3.28) | 4.71 (4.66-4.75) | 3.29 (3.25-3.32) | 4.49 (4.45-4.54) | 2.93 (2.9-2.96) | 5.3 (5.24-5.35) | 3.46 (3.42-3.49) |
| 2 | 4.77 (4.72-4.81) | 3.25 (3.21-3.28) | 4.81 (4.76-4.86) | 3.27 (3.23-3.3) | 4.68 (4.63-4.73) | 2.96 (2.93-3) | 5.4 (5.35-5.46) | 3.43 (3.39-3.46) |
| 3 | 4.87 (4.82-4.92) | 3.3 (3.26-3.33) | 4.97 (4.92-5.02) | 3.36 (3.32-3.39) | 4.87 (4.82-4.92) | 3.07 (3.04-3.1) | 5.62 (5.56-5.67) | 3.54 (3.51-3.58) |
| 4 | 4.91 (4.86-4.96) | 3.29 (3.26-3.32) | 4.98 (4.93-5.03) | 3.32 (3.29-3.36) | 4.88 (4.83-4.93) | 3.04 (3.01-3.07) | 5.69 (5.64-5.75) | 3.55 (3.51-3.58) |
| 5 - most deprived | 5.24 (5.19-5.29) | 3.42 (3.39-3.46) | 5.18 (5.13-5.23) | 3.38 (3.34-3.41) | 5.04 (4.99-5.09) | 3.06 (3.03-3.09) | 5.72 (5.66-5.78) | 3.48 (3.45-3.52) |
| Face-to-face | | | | | | | | |
| 1 - least deprived | 3.67 (3.63-3.71) | 2.6 (2.57-2.63) | 3.76 (3.72-3.8) | 2.66 (2.63-2.69) | 2.17 (2.15-2.2) | 1.45 (1.44-1.47) | 3.04 (3.01-3.08) | 2.02 (2-2.04) |
| 2 | 3.87 (3.83-3.9) | 2.66 (2.63-2.69) | 3.9 (3.86-3.94) | 2.68 (2.65-2.71) | 2.3 (2.28-2.32) | 1.49 (1.48-1.51) | 3.12 (3.09-3.16) | 2.01 (1.99-2.03) |
| 3 | 3.91 (3.87-3.95) | 2.67 (2.65-2.7) | 3.98 (3.94-4.02) | 2.72 (2.69-2.75) | 2.37 (2.34-2.39) | 1.53 (1.51-1.54) | 3.24 (3.21-3.28) | 2.08 (2.05-2.1) |
| 4 | 3.95 (3.91-3.99) | 2.68 (2.65-2.7) | 3.99 (3.95-4.03) | 2.7 (2.67-2.73) | 2.3 (2.27-2.32) | 1.47 (1.45-1.48) | 3.25 (3.21-3.28) | 2.06 (2.04-2.08) |
| 5 - most deprived | 4.25 (4.21-4.29) | 2.81 (2.78-2.84) | 4.2 (4.16-4.24) | 2.77 (2.74-2.8) | 2.29 (2.26-2.31) | 1.42 (1.41-1.44) | 3.23 (3.2-3.27) | 2 (1.98-2.02) |
| Remote | | | | | | | | |
| 1 - least deprived | 0.92 (0.9-0.93) | 0.6 (0.59-0.6) | 0.9 (0.89-0.91) | 0.58 (0.57-0.59) | 2.28 (2.25-2.3) | 1.45 (1.43-1.47) | 2.23 (2.2-2.26) | 1.42 (1.4-1.44) |
| 2 | 0.85 (0.84-0.86) | 0.54 (0.53-0.54) | 0.86 (0.85-0.88) | 0.54 (0.53-0.55) | 2.34 (2.32-2.37) | 1.44 (1.43-1.46) | 2.26 (2.23-2.29) | 1.4 (1.38-1.42) |
| 3 | 0.91 (0.9-0.93) | 0.57 (0.56-0.58) | 0.94 (0.93-0.95) | 0.58 (0.58-0.59) | 2.46 (2.43-2.49) | 1.51 (1.49-1.53) | 2.36 (2.33-2.39) | 1.45 (1.43-1.47) |
| 4 | 0.92 (0.9-0.93) | 0.56 (0.55-0.57) | 0.96 (0.94-0.97) | 0.58 (0.57-0.59) | 2.56 (2.53-2.59) | 1.54 (1.52-1.56) | 2.44 (2.41-2.47) | 1.47 (1.45-1.49) |
| 5 - most deprived | 0.94 (0.93-0.95) | 0.57 (0.56-0.57) | 0.94 (0.93-0.95) | 0.56 (0.55-0.57) | 2.73 (2.69-2.76) | 1.61 (1.59-1.63) | 2.49 (2.46-2.52) | 1.47 (1.45-1.49) |
